# The Role of Lichens, Mosses, and Vascular Plants in the Biodeterioration of Historic Buildings: A Review

**DOI:** 10.3390/plants11243429

**Published:** 2022-12-08

**Authors:** Alessia Cozzolino, Paola Adamo, Giuliano Bonanomi, Riccardo Motti

**Affiliations:** 1 Department of Agricultural Sciences, University of Naples Federico II Via Università, 100, 80055 Portici, Italy; 2 Interdepartmental Research Centre on the ‘Earth Critical Zone’ for Supporting the Landscape and Agroenvironment Management (CRISP), University of Naples Federico II, 80055 Portici, Italy; 3 Task Force on Microbiome Studies, University of Naples Federico II, 80138 Naples, Italy

**Keywords:** biodeterioration, monument conservation, higher plants deterioration, biodeteriogenic plants, hazard index

## Abstract

Biodeterioration is defined as the alteration of a given substrate due to a combination of physical and chemical factors produced by living organisms when attached to such materials. This phenomenon attracts scientific research attention due to its risk in causing destruction to outdoor cultural rock heritage sites. In this review, an update on the state-of-art regarding the biodeterioration phenomenon is represented in order to highlight the type of colonizing vegetation and possible mechanisms behind the corresponding deterioration. For this reason, 62 articles with a focus on lichens, mosses, and higher plants were investigated by evaluating the role of construction materials and different plant species related to the hazard index. The results showed that trees and shrubs are the most harmful plant life forms, for example, *Ficus carica*, *Ailanthus altissima*, and *Capparis spinosa*, while regarding building materials, those characterized by high porosity, such as andesite and argillaceous limestone, are more vulnerable to plant colonization. Further studies are needed to examine in detail the relationship between colonizing organisms, intrinsic elements of the substrate, and external factors, as well as the refinement of measures to prevent and control colonization by plants.

## 1. Introduction

Biodeterioration is a type of damage generated by the growth and/or metabolic activities of organisms on various substrates. As defined by Hueck [1], biodeterioration is “any undesirable change in the properties of a material caused by the vital activities of organisms”, or, as defined by Rose [2], it is “the process by which living organisms are the cause of the [structural] loss of quality and, consequently, of value”. This definition separates the concept of biodeterioration from cases of wear and corrosion involving undesirable changes in the properties of a material caused by mechanical, physical, and chemical influences [3].

Historical buildings and stone monuments are the maximum expression of human heritage and, being subject to deterioration, their conservation has become a matter of concern for the scientific community to ensure the identity and cultural continuity of humanity [4,5].

Therefore, understanding the physiological and morphological characteristics of deteriogenic organisms, such as algae, fungi, lichens, mosses, and vascular plants, is necessary to establish the type of interaction that occurs with the material and to evaluate the cause–effect of the deteriorating action of a specific biological agent [6]. The colonization of stone monuments by an organism also depends on the chemical–physical properties of a material, giving rise to a particular susceptibility to colonization, defined as bioreceptivity, which is based on the surface roughness, porosity, exposure, and inclination of the wall [5,7,8,9]. Obviously, the building materials are more or less bioreceptive and therefore subject in different ways to biological degradation.

Environmental conditions such as moisture, wind, and temperature can play a crucial role in the colonization and establishment of living communities that can affect the stone surfaces of monuments and works of art. As the alteration processes proceed, living organisms begin to colonize the area and so the phenomenon of biodeterioration takes place [10]. Several authors have reported that microorganisms can play a significant role in all rock deterioration processes, developing on the stone surface even when no organic matter is present [11]. Algae and cyanobacteria are usually the first colonizers due to their limited water demands, then lichens follow, which are highly resistant to drying and high temperatures, and fungi, which cause alterations (e.g., stains and loss of cohesion) that are difficult to identify by direct observation [12,13]. With a persistent deteriogenic action of environmental agents and microorganisms, the colonization of buildings by vascular plants is favored and increased.

Recently, many studies have been investigating the role played by vascular plants in causing damage to stone monuments in Mediterranean bioclimatic conditions, in temperate bioclimates, and in tropical or subtropical areas [14].

This review aims to contribute to the knowledge of the state-of-the-art of the biodeterioration phenomenon on historical buildings, describing the succession of living organisms that takes place during the process. We focused on lichens, mosses, and higher plants by investigating aspects that in the existing literature have been studied mostly for fungal and bacterial organisms [15,16]. Biotic and abiotic factors affecting colonization of the organisms under study were analyzed, reporting indications of the various control methods to prevent the spread of biodeterioration.

## 2. Biodeterioration Process

Deterioration occurs after a strong alteration due to weathering and aging of stone materials. Weathering is a combination of geochemical, geophysical, and biological processes responsible for the alteration of source rocks.

Physically, rock masses are eroded from an overlying rock resulting in fragmentation of the outcrop, aggravated by temperature differences which consequently emphasize the susceptibility to chemical attack [17]. Chemical weathering changes the chemical composition and structure of source rocks, leading to disintegration and in turn increasing the mineral surface area available for chemical weathering [18]. Bioweathering, instead, consists of the alteration of rock-forming minerals by biota (microorganisms, plants, and animals) which produce mechanical forces and metabolic compounds, including chelators and organic acids [17]. Generally, biodeterioration can be classified in three different types: physical or mechanical, aesthetic, and chemical. The physical type is related to the processes of growth and the movement of living organisms which destroy the material structure (e.g., root damage, gnawing by rodents). From an aesthetic point of view, the most significant effects depend on encrustation or soiling due to the presence of organisms, their metabolic products, and/or dead bodies, which form a microbial layer on the surface known as biofilm. Finally, chemical biodeterioration includes two different processes: assimilatory and dissimilatory. In the process of assimilation, organisms use the structural component as a food source by changing the properties of the material, while in the dissimilatory process, organisms excrete waste products or other substances that negatively affect the material [19,20].

As autotrophic organisms, algae and Cyanophyta are typically deemed the first microorganisms to colonize historical artifacts. Their presence is indicated by the deposit of a patina which can cause chemical or mechanical stone degradation [21]. Cyanophyta and other N-fixing organisms along with more complex communities, such as lichens, produce materials that increase the amount of nitrogen and can facilitate the establishment of rootless plants, such as mosses. Thus, in the early stages of colonization where the fixed nitrogen is a limiting nutrient, with the main reserve of nitrogen in the biosphere being molecular di-nitrogen, biological nitrogen fixation is among the first chemical mechanisms related to substrate formation. It consists of the conversion of atmospheric nitrogen into ammonia by symbiotic, associative, and free-living bacteria [22,23]. Then, microbe and lower plants release secretions which play a pioneering role in soil formation accelerating the process of rock weathering and altering the properties of parent material. A constant secretion production gives rise to a shallow, original soil, which is being enriched by nutrients due to biological growth that limits leaching processes. At the same time, the strong interaction between the non-vascular vegetation and the original soil allows a further accumulation in nutrients available to higher plants, whose activity promotes the production of fertile soil [17,24].

**Figure 1:**
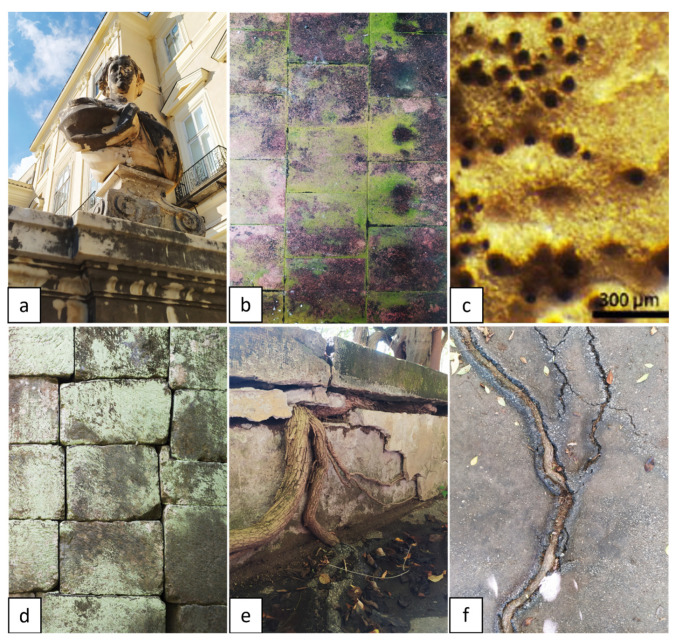
Increasing damage caused by colonizing organisms: (**a**) black crust caused by heterotrophic bacteria (Royal Palace of Portici, Naples, Italy); (**b**) green patina caused by cyanobacteria (Royal Palace of Portici, Naples, Italy); (**c**) bio-pitting caused by lichens [25]; (**d**) discoloring caused by mosses (Royal Palace of Portici, Naples, Italy); (**e**) cracks in the wall caused by vascular plants (Botanical Garden of Portici, Naples, Italy); and (**f**) cracks in the pavement caused by vascular plants (Botanical Garden of Portici, Naples, Italy). Pictures by Alessia Cozzolino.

Hence, pioneer microorganisms and plants (both therophyta and hemicryptophyta) are always present in the colonization process and can cause minimal damage, with them being replaced later by more harmful plants such as small shrubs or trees (Figure 1).

**Figure 2:**
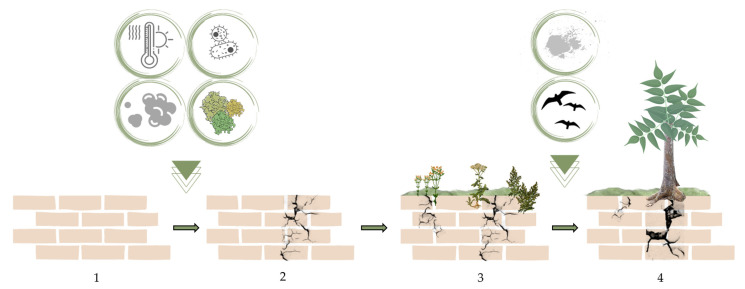
Wall colonization stages: abiotic and biotic factors cause a fracture (**1**,**2**) in which a thin layer of soil accumulates creating the necessary conditions for herbaceous plants to thrive (**3**). Subsequently, colonization by arboreal plants (**4**) occurs due to the presence of a large layer of soil and other elements accumulated in the fracture that have grown in size over time.

**Figure 3:**
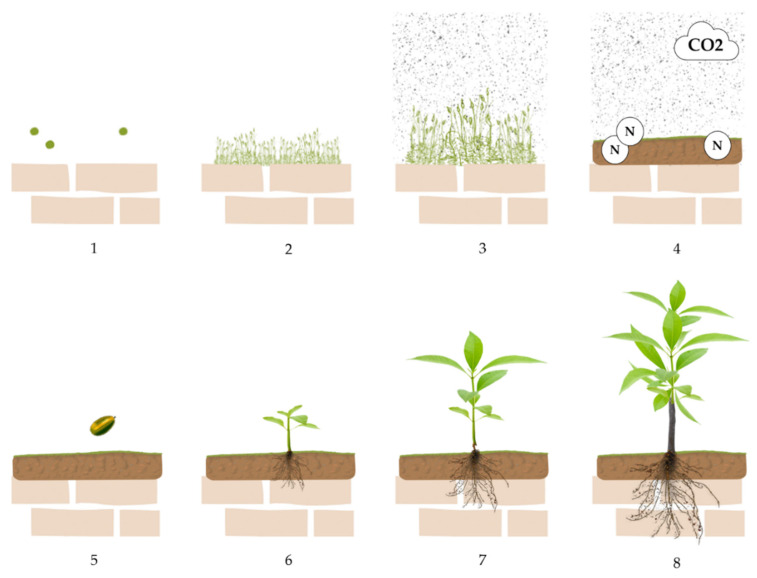
Wall colonization stages with pioneering moss: moss spores fall on porous stone and develop (**1**,**2**). The storage of atmospheric dust leads to the creation of substrates (**3**,**4**) that can host a seed of an herbaceous plant (**5**). The plant grows (**6**,**7**) and damages the wall with the expansion of the root system (**8**).

Considering this sequence, two colonization modes can be described in relation to exposure, slope, water availability, and other factors that influence the arrival of some organisms over others depending on their adaptation to the present conditions. In the first, there is the intervention of abiotic factors that create the necessary conditions for the development of bacteria, fungi, and lichens. The latter deteriorate the surfaces causing the formation of a substrate suitable for the first pioneer plants (i.e., *Sonchus tenerrimus*, *Parietaria diffusa*) which in turn form a favorable substrate for other higher plants through their detritus, atmospheric dust, bird excrement, and human wastes (Figure 2). The second mode of colonization can occur where there are horizontal surfaces and there is good water availability, so the pioneer organisms can be mosses that trap atmospheric dust leading to the formation of substrates necessary for the germination of seeds of vascular plants (Figure 3).

### 2.1. Focus on Lichens

**Figure 4:**
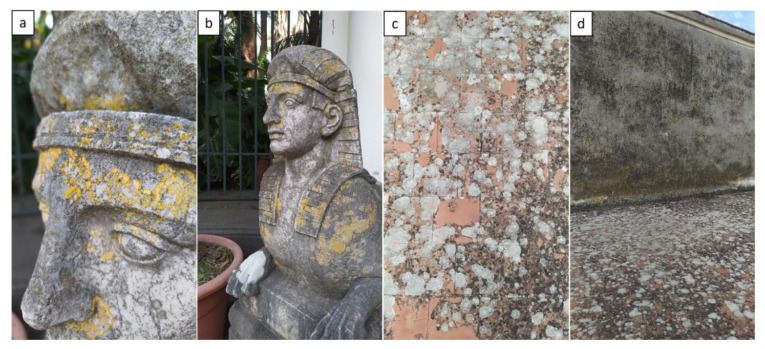
Lichens on statues representing sphinxes at the Botanical Garden of Portici (Naples, Italy) (**a**,**b**); and lichens on the roof of the Royal Palace of Portici (Naples, Italy) (**c**,**d**). Pictures by Alessia Cozzolino.

The succession of communities of living organisms growing on stone artifacts generally begins with the development of lichen flora depending on organic contamination caused by moisture and wind on the stone over time [21]. Lichens occupy environments normally hostile to many other forms of life, perhaps due to their resistance to desiccation and extreme temperatures, to longevity, and to their ability to store nutrients from the surrounding environment (Figure 4) [26].

They have a simple structure known as thallus, consisting of a symbiotic union of a fungal element, the mycobiont (Ascomycetes or seldom Basidiomycetes), and a photosynthetic organism (cyanobacteria or green algae), the photobiont. The upper side of the thallus is covered by a cortical layer formed by organized hyphae, and besides the algal zone there is the medulla, which consists of interwoven hyphae [13,27].

The colonization of stone materials by lichens is favored by the wind, insects, and birds which disperse the spores in the atmosphere which, falling into pores or cavities with water, favors the development of new organisms [13]. According to Garcia-Rowe and Saiz-Jimenez [26], the large-bird excrement provides the organic nitrogen useful to the develop of an ornithocoprophilous flora, which, in the long term, can cause biodeterioration of materials. Indeed, on the cathedral of Salamanca (Spain) there are some species of lichens, such as *Ramalina* spp., which are characteristic of rainy climates and excrement-rich habitats [26].

The damage caused by lichens is both mechanical and chemical. The first is due especially to the penetration of the hyphae into the substrate, causing loss of cohesion due to the contraction and expansion of the thallus during fluctuations in the water supply. Chemical damage, instead, regards the production of organic carboxylic acids, such as citric, oxalic, lactic, and gluconic acids which favor the chemical processes by which the lichens are able to decompose lithic constituents [13,27].

On the other hand, a bioprotective condition also occurs. For example, in the case of sandstone, the oxalic acid can react with the calcite cement producing calcium oxalate crystals around the lichen hyphae. When the thallus dies, the union of oxalates and organic and mineral matter form a patina which provides good protection to the sandstone against weathering. In agreement with Arino et al. [28], this balance between biodeterioration and bioprotection was observed at the forum of Baelo Claudia, a Roman city located in southern Spain. The flagstones without lichen cover showed higher deterioration than those colonized by lichens thanks to several preventive actions: reduction of the abrasive effect produced by airborne sand particles, reduction of the dissolution effect of water lying on the surface, reduction of temperature changes, etc. [28].

Furthermore, some authors argue that when the growth of lichens does not damage a monument, their presence can even enrich its cultural value by strengthening its historical and artistic importance [13].

### 2.2. Focus on Mosses

**Figure 5:**
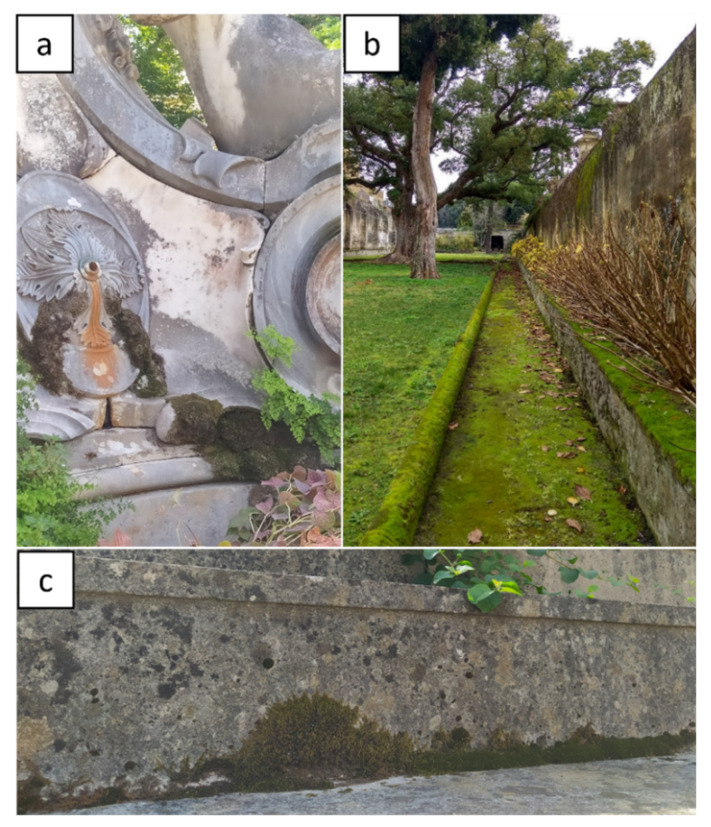
Mosses in the Botanical Garden of Portici (Naples, Italy): (**a**) “Fontana della Vittoria”; (**b**) the Secret Garden; and (**c**) steps. Pictures by Alessia Cozzolino.

With a sufficient accumulation of soil in the micro-cavities of rocks, the development of mosses and liverworts can begin [21]. Mosses generally prefer wetter climates in which humid environmental conditions and low temperature promote their regeneration and multiplications (Figure 5). Thus, they retain water and abundant nutrients providing favorable environments for microorganism growth, but consequently, the accumulation of moisture promotes the deterioration and weathering processes of building materials [29,30,31].

Focusing on dry climates, most dryland mosses have evolved to require desiccation by losing virtually all liquid water from their shoot tissues [29,32]. According to Jang and Viles [29], the mosses have a buffering effect that may be beneficial to buildings located in drier climates. This result was observed at the churchyard at Chipping Norton where the comparison between bare stone and moss-covered stone showed that the mosses can shield the substrates from lighter periods of rain and moisture. So, for built heritage sites in these climates, the removal of mosses may not be necessary for the health and preservation of the site. Even, additional tests (e.g., Karsten tube tests) illustrated that the impact of removing mosses may be more damaging than leaving them in situ.

Conversely, in wetter climates and in cases with dramatic and invasive growths, different methods are implemented to preserve and control relics: direct methods (mechanical, biological, chemical, and physical) to counter the microorganisms; and indirect methods to confine the microorganisms’ activity, thus eliminating or reducing their nutritional sources [29,30].

Removing mosses therefore implies a dramatic effect on the aesthetics and presentation of a cultural heritage asset because their presence may be beneficial to the appearance of the monuments as a result of a higher authenticity value. This is the case of Dryburgh Abbey where weathering and decay can accrue ‘age value’, marking the passage of time and creating aesthetically pleasing ‘character’, ‘patina’, and ‘ruination’ [29,33].

### 2.3. Focus on Higher Plants

Vascular plants’ colonization of stone artifacts is limited by the availability of suitable sites for the settlement, the type of substrate, the disturbance, and the variability of the microclimate in terms of moisture and temperatures [34,35,36].

Stone artifacts are a harsh environment for plant growth and, as underlined by Segal [34], they show ecological characteristics similar to those of rocks in natural environments and can be considered selective anthropic ecosystems [37,38]. The structural and environmental features of walls affect their ability to act as suitable habitats for plant growth. The factors that most influence the capacity of the walls to serve as habitats for higher plants are the size, construction materials, position, and age [38]. The settlement of vascular plants usually occurs in crevices and fractures in the wall, and if left undisturbed, a succession takes place of plants of increasing diversity and size [3,38]. Vascular plant colonization is essentially conditioned by the adaptability of the species and the efficiency of their mode of reproduction. The living organisms mentioned above are the most harmful biodeteriogens, causing cracking and disconnection of materials, leading to considerable problems for stability and safety. From a biophysical point of view, the decay is mainly caused by the roots, as the radial thickening during growth causes higher pressure on the structural parts of the building. The roots develop in fissures or cracks already present in the walls, in water seepage, and in more fragile parts (e.g., mortar between stones) by reducing the adhesion between the stones [13,39]. In addition, soil forms in the cracks creating niches favorable to the accumulation of nutrients and organic substance, which encourage the development of characteristic vegetation of the biogeographic area affected [34,37]. With regard to the biochemical processes, a dualism can be noted between a mechanism of assimilation where the organism obtains nourishment by exploiting the surface of stones and a mechanism of dissimilation in which the production of metabolites by organisms causes chemical reactions on building materials [37,40,41].

The type of damage that occurs on monuments varies in relation to the biological form of the plants or the characteristics of the root system [14]. Plant biological form has been considered by most authors as the main element for assessing the hazard risk [3,35,42,43]. There are different categories of plants, based on their life cycle and the position of the buds: therophytes-T: annual herbs (short life of few months); hemicryptophytes-H: perennial herbs with buds at the soil level; geophytes-G: perennial herbs with underground storage organs; chamaephytes-Ch: woody plants with buds at no more than 25 cm above the soil surface; and phanerophytes-P: trees and shrubs with buds over 25 cm above the soil surface [44]. After classifying the biological form, it is possible to evaluate the hazard of deteriogenic species according to Signorini [42], assigning a numerical index, defined as a hazard index (HI), ranging from 0 (minimal hazard) to 10 (high hazard). It differs for each species and is based on plant life form, invasiveness, vigor, and the size and shape of the root systems. Moreover, if there is visible damage, the risk assessment method proposed by Fitzner and Heinrichs [45] can be followed, which considers the recurrent damaging phenomena due to plant colonization, such as detachments, cracking, and deformation [14,35,37].

**Figure 6:**
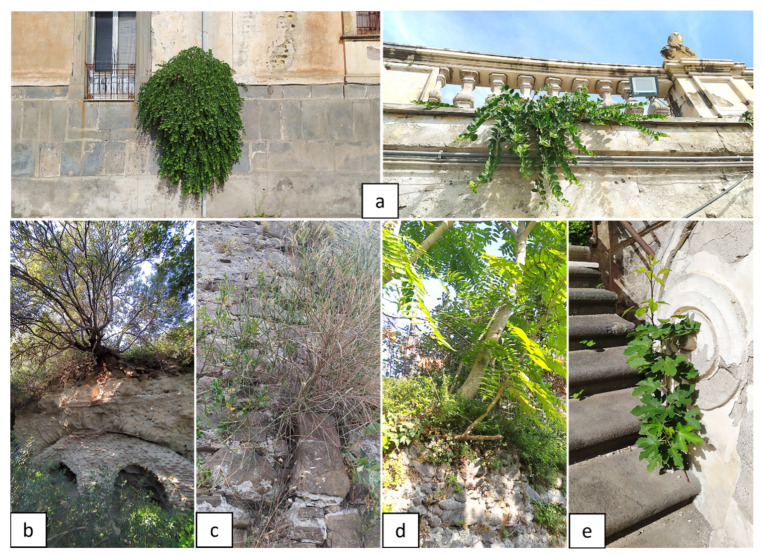
Harmful biodeteriogens species: (**a**) *Capparis orientalis* (Royal Palace of Portici, Naples, Italy); (**b**) *Pistacia lentiscus* (Archaeological Park of Pausilypon, Naples, Italy); (**c**) *Spartium junceum* (Agropoli Castle, Salerno, Italy); (**d**) *Ailanthus altissima* (Somma Vesuviana, Naples, Italy); (**e**) *Ficus carica* (Royal Palace of Portici, Naples, Italy). Pictures by Alessia Cozzolino.

Generally, the phanerophytes have the highest impact with an average HI of 7, since their shape and characteristics make them more harmful. Then, there are the therophytes (1.1), geophytes (3.1), chamaephytes (4.3), and the hemicryptophytes (4.3) [3]. According to several authors, the most common trees and shrubs with the higher HI among the species are *Ailanthus altissima* (HI 10), *Celtis australis* (HI 10), *Ficus carica* (HI 10), *Capparis orientalis* (HI 10), *Quercus ilex* (HI 9), *Cytisus infestus* (HI 8), *Spartium junceum* (HI 8), *Sambucus nigra* (HI 8), *Pistacia lentiscus* (HI 8), etc. (Figure 6) [3,8,36,37].

**Figure 7:**
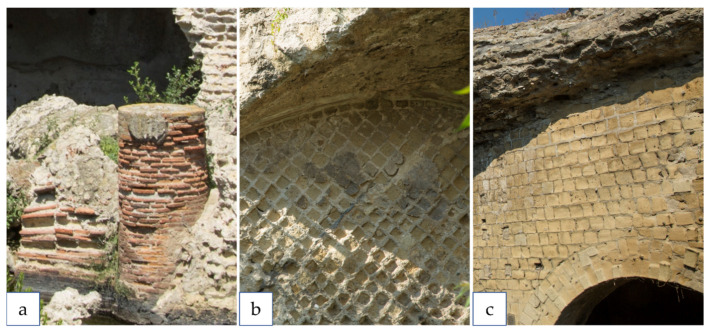
Common substrates found in the PFAP: (**a**) Opus latericium, (**b**) Opus reticulatum, (**c**) and yellow tuff. Pictures by Alessia Cozzolino.

The floristic composition in the various historical sites is related to the type of construction material in association with exposure, inclination, and surrounding environmental characteristics, including human disturbance. According to Motti et al. [37], in the Phlegraean Fields Archaelogical Park (PFAP), located in the province of Naples (southern Italy), all the major deteriogenic higher plants grow on more or less porous construction materials such as yellow tuff, bricks, and conglomerate, with the average plant cover considerably higher on vertical surfaces and at western and southern exposure (Figure 7). In fact, the vertical surfaces show a higher abundance of species with the highest hazard index (HI > 5), such as *Artemisia arborescens*, *Rubus ulmifolius*, *Reichardia picroides*, *Capparis orientalis*, *Pistacia lentiscus*, *Matthiola incana,* and *Ficus carica*, while *Ailanthus altissima* grows almost exclusively over horizontal substrates. Regarding the substrate, some species (e.g., *Rubus ulmifolius* and *Rhamnus alaternus*) are not affected by the type of material, unlike other species, such as *Artemisia arborescens*, *Matthiola incana*, *Spartium junceum,* and *Ailanthus altissima,* which mostly grow on yellow tuff. So, based on the observations, herbaceous species colonize low porous lithotypes such as basalt, mosaic, and marble, while tree species grow preferably on volcanic rocks and materials characterized by a strong porosity that helps to retain more water. Moreover, woody plants (phanerophytes) are present mostly on western and eastern exposure; in contrast, herbaceous species (hemicryptophytes and therophytes) prefer the south-facing slopes.

Hosseini et al. [14] surveyed the monuments of the Pasargadae World Heritage Site in (Iran) to identify the substrate preference of plants in colonizing stone surfaces. There are two types of limestones called beige stone (BS), a pure aspartic limestone composed by calcite and dolomite, and green-gray stone (GGS), an argillaceous limestone small quartz and non-swelling clay minerals. Some species showed a clear preference for GGS: *Glycyrrhiza glabra*, *Senecio glaucus*, *Crepis sancta*, *Euphorbia dracunculoides*, *Poa bulbosa*, *Medicago persica*, *Lepidium draba*, *Lactuca serriola*, *Tragopogon graminifolius,* etc. Other species, instead, showed propensity for BS: *Adonis aestivalis*, *Peganum harmala*, *Papaver argemone*, *Scandix stellata*, *Euphorbia sororia*, *Ficus johannis*, and *Nonea longiflora*. On the basis of the various observations, a high number of species with a low abundance were observed on Beige stone; conversely, on Green-Gray stone, the number of species with a low abundance was particularly lower and species with a higher abundance were more numerous, which proved its suitability for plant colonization.

Another study to evaluate the relationship between plant biodiversity and exposure and building materials was carried out for three historical Calabrian (Southern Italy) churches by Mascaro et al. [8]. The facades of Santa Maria della Serra and Santissima Annunziata consist of white-yellowish or reddish fossiliferous calcarenites, while Santa Maria della Pietà is composed of carbonate rock. Among the 27 species recorded, 16 were present on vertical surfaces, all Geophytes and chamaephytes on horizontal surfaces, while therophytes were instead on vertical surfaces. *Parietaria judaica* was the only species present at all three sites. Regarding the hazard index values, only the site of Santa Maria della Pietà presented a high average value of HI (HI 6.3) due to the presence of *Ailanthus altissima* (HI 10), *Ficus carica* (HI 10), *Rubus ulmifolius* (HI 8), and *Sambucus nigra* (HI 8). At any rate, the colonizing species of the three sites suggested that the different substrates did not influence the growth of plants. Moreover, although previous studies have shown that most plants dwell on horizontal surfaces for the best growing conditions, in the case of the Calabrian churches, more species have been observed on vertical surfaces, which is in agreement with Motti et al. [37]. Nonetheless, in contrast with the abovementioned case study, therophytes were more common on horizontal surfaces, while geophytes and chamaephytes were on vertical surfaces.

With reference to surface roughness, we can again mention the work performed by Korkanç and Savran [21]. In the examined historical buildings of the Niğde region, in the horizontal or almost horizontal position, a material with high surface roughness and high porosity was used. This condition simplified the formation of soil and succession on the stone, especially in the areas facing north and the moist parts of the construction.

It is actually in these microsites that birds and/or other organisms (e.g., ants) prefer to transport seeds during seasons characterized by high temperatures. The hemicryptophytes have been largely checked on the stone artifacts, and the seedy plants that were disseminated more involved: *Ajuga chamaepitys*, *Verbascum sinuatum*, *Stipa holosericea*, *Noae mucronata*, *Sanguisorba minor*, *Descuriana sophia*, *Mercuralis annua*, *Scorzenora mollis*, *Scrophularia libanotica*, *Rubia tinctorum*, *Parietaria lusitanica*, *Alkanna orientalis*, *Alkanna tinctoria*, *Euphorbia macroclada*, *Isatis glauca*, *Lactuca scariola*, *Poa bulbosa*, *Scandix stellata*, *Galium verum*, *Lamium amlexicaule*, *Lepidium perfoliatum*, *Veronica triphyllos*, *Reseda lutea*, *Asperugo procumbens,* and *Bromus tectorum*. After the growth of herbaceous plants, some habitats, in particular the roof section made of andesite, have improved and changed to host even small woody species including *Capparis spinosa*, *Thuja orientalis*, *Berberis crataggina*, *Cerasus avium*, *Prunus armenica,* and *Prunus domestica*.

## 3. Factors Affecting Biodeterioration

There are numerous ecological factors, such as physical, chemical, or biological factors, that condition the growth and the life of an organism, following generally two laws underlying ecology.

“Liebig’s law of the minimum” (1840) governs nutrient limitation, stating that in conditions of stationary equilibrium, the essential substances available in quantities close to the minimum necessary tend to become limiting (Van der Berg) [46]. At the same time, an environmental factor may represent a minimum or maximum limit for a species, determining the tolerance of the species to this factor. This is the Shelford’s law (or the law of tolerance, 1913) considered as an extension of the previous one, since not only the minimum value but also the maximum value may be a critical factor [39,47].

Generally, the most relevant environmental factors for the growth of an organism are water, temperature, light, and nutrients, because their values are often close to the minimum limit for the survival of the species. This explains why climatic factors play a primary role in biodeterioration processes [39].

Water accumulating in buildings is among the main causes of decay [48]. Its availability is a fundamental parameter which determines the initiation of microbial susceptibility and colonization, biofilm formation, and subsequent biological degradation. Moisture content in building stones is the result of a dynamic equilibrium between the material and the environment, and it is influenced by the movement of water that goes from inside to outside of the masonry and vice versa [49,50]. Water can reach building materials in several ways, from driving rain, capillary water rising from underground, run-off from the roof, and condensation of air humidity. Capillary rise is the main mechanism for water to infiltrate a building material, hence capillary water is very important for the establishment of microorganisms [48,49,51]. Moisture acts as a substrate for the growth of living organisms such as bacteria, fungi, or algae with consequent physical and chemical damage. For these reasons, it is important to find prevention strategies to avoid problems related to humidity, since eliminating the already existing problem is complicated, especially in structures of historical value [48].

Other important factors involved in the deterioration of stones are related to the substrates and construction materials, from a physical (roughness and porosity) and chemical (mineral composition and surface pH) point of view. In this regard, it is useful to mention the concept of biorecectivity proposed by Guillitte [9], considered as “the totality of material properties that contribute to the establishment, anchorage, and development of fauna and/or flora” or as “the aptitude of a material to be colonized by one or several groups of living organisms without necessarily undergoing any biodeterioration”. This concept originates from the term “susceptibility” and involves an ecological correlation between the colonizer and the substrate. Materials with high porosity have a high bioreceptivity index (BI), as more water is retained inducing worsening of the deterioration process [9,52]. Although a standard laboratory protocol for estimating stone biocerectivity and defining its index has not yet been established, Vázquez-Nion et al. [53] proposed a BI for granitic rocks including two components: *BI_growth_* that quantifies the extent of the biological growth and *BI_colour_* that quantifies the color change undergone by the stone due to the colonization. BI values are adapted to a scale that allows the qualitative classification of lithotypes in a simple and easily comprehensible form.

Bioreceptivity cannot be described as a static property, rather it varies for each stone material according to different stages of deterioration. Thus, Guillitte [9] defined three types of bioreceptivity: “primary or intrinsic bioreceptivity”, which is related to the initial potential of biological colonization of sound stone, “secondary bioreceptivity”, which refers to the potential of biological colonization of weathered stone, and “tertiary bioreceptivity”, which is the colonization potential of a stone material subjected to conservation treatments [9,11].

Some researchers think that roughness has the most significant influence of all the properties. Firstly, microorganisms and organic materials that are transported to the building by wind and water adhere to the substrate. Then, especially when the substrate color is lighter, the surface roughness is directly proportional to the adsorption of solar radiation, thereby influencing the surface temperature [21]. In a study conducted by Korkanç and Savran [21], the mineralogical and engineering properties of the stones used in six historical buildings located in central Anatolia (Niğde region) were determined to describe the great impact that these characteristics have on plant growth. According to the data obtained, ignimbrites showed the highest water absorption and porosity rates and the highest surface abrasion values. Tuff showed higher compressive strength values than ignimbrites, but lower abrasion and compressive strength values were recorded for fine-grained tuffs. Travertines showed high abrasion loss and low compressive strength, while fine-grained travertines showed lower porosity and water absorption and higher abrasion and compressive strength values. Andesites presented higher compressive strength values than other volcanic rocks (Table 1). With regard to surface roughness, the highest values were found in travertines with high porosity, as well as the low-welding ignimbrites and tuffs with a considerable amount of coarse rock fragments (Table 2).

So, based on the studies performed, the lowest engineering properties were determined for ignimbrites and tuffs, and the major biodeterioration effects were observed on travertine, ignimbrite, and andesite, which have high surface roughness.

## 4. The Most Current Strategies to Counter the Phenomenon

The control of biodeterioration processes should begin with preventive measures that prevent the formation of favorable conditions for the development of deteriogenic microflora. Referring to Liebig’s law of the minimum (1840), in order to avoid or control a biological attack on a material, the limiting factor could be identified to modify or reduce its values to a level below the limit necessary for growth. It is not necessary that all parameters for the survival of the species are brought below the minimum level, but it is sufficient that at least one of these values is modified to obtain an inhibitory effect on growth [39]. Furthermore, considering that water is one of the most influential factors in the biodeterioration process, the reduction of moisture within the stone material is necessary, by optimizing drainage systems, correcting architectural defects, and applying protective treatments to the stone. Nevertheless, the preventive actions are not always sufficient [10]. Considering building materials, biodeterioration can play a significant role in the life cycle analysis of large infrastructure systems [19]. So, proper knowledge of environmental conditions, building materials, and colonizing organisms enables the development of new technologies and safer and more effective conservations treatments. Over the centuries, biodeterioration has been treated with mechanical procedures, such as sand blasting, air abrasion, steam cleaning, pressure washing, oils, or even wax. However, these physic-mechanical treatments, which interact with the stone, are often not adequate for historical building as they are very aggressive. In recent times, new methods and eco-friendly or low impact procedures are being perfected to reduce the health risks for the individuals involved in restoration, visitors, and the environment. These methods incorporate both cleaning procedures to remove patinas and biocidal treatments to kill colonizing organisms [54,55]. Before using biocides, effective control of water and nutrient availability by and in the material is required; in fact, biocides should only be applied where biodeterioration cannot be controlled and chemical interventions are unavoidable [10]. Generally, to lower the required concentrations of various type of biocides, a first step in the design of more ecologically friendly solutions may be pre-treatment with water-repellent coatings. Moreover, a detailed analysis of the microbial composition colonizing building materials and a species- and site-specific calibration of biocidal strategies is necessary to select the best biocide or coating to use, thus avoiding non-essential compounds and their release into the environment [55,56,57]. A good solution to improve the efficiency of biocide treatments and to avoid the environmental risks associated with their possible toxicity was studied by Zuena et al. [58]. The researchers applied on four different type of stones (brick, mortar, travertine, and Carrara marble) a novel coating charged with an eco-friendly biocide, the zosteric sodium salt, encapsulated into two silica nanocontainers and dispersed into a tetraethoxysilane-based (TEOS) coating also containing TiO_2_ nanoparticles. This formulation showed hydrophobic and biocide properties, based on the reduction in the capillary water absorption coefficient and the decrease in microorganism adhesion on cell surfaces at non-toxic concentrations, respectively.

Referring to lichens, for foliose and fruticose ones, the mechanical removal of the lichen thalli is performed as they do not adhere strongly to the substrate. For crust-like lichens, instead, the removal of the thallus which penetrates deeply into the stone requires the use of hard brushes and considerable washing with water and detergents.

However, this method causes the dispersion of spores and increased stone loss, so it becomes necessary to employ biocides. The application of these products is performed by spraying, avoiding windy and rainy days, or brushing, preferably on non-friable surfaces. Several authors recommend the use of quaternary ammonia salts applied with a low-pressure spray, choosing the correct concentration in relation to the porosity of the substrate [14].

In the present situation, no “gold standard methodology” has yet been established. Indeed, Romani et al. [55] recommend addressing the problem of biodeterioration on a case-by-case, adopting a multidisciplinary research strategy, utilizing the skills of biologists, chemists, and material designers. This innovative solution was successfully designed without mechanical impact to treat temples in Angkor Vat (Cambodia) [59] and ruins of the archaeological site of Milet (Turkey), where a black microbial biofilm needed to be removed as it enhanced the heating effect leading to microcracking of the marble [60].

While damage caused by micro-organisms is often hardly noticeable, damage caused by vascular plants is more visible and severe, depending on the extent of the growth rate and biological form of the plant. Decay is caused by the aerial part, but mainly by the roots, as they deepen into the structure and grow to a large size, causing physical and chemical issues. Roots secrete substances that attack building materials, and in the course of their growth, they open cracks, cause crumbling, and loosen stones and large fragments in the wall [13]. According to Almeida et al. [61], trees and shrubs such as *Ailanthus altissima*, *Capparis spinosa*, *Clematis vitalba*, *Ficus carica*, *Hedera helix,* and *Rubus ulmifolius* are typically more destructive than herbaceous plants such as *Mercurialis annua*, *Parietaria diffusa,* and *Sonchus tenerrimus.* Aesthetically, *Hedera helix* is very attractive, but creates many difficulties: the fruit is eaten by birds and the seeds germinate in many microsites; if the plant grows as a liana, the weight damages the surfaces; if the plant germinates and grows in the wall, there is additionally the destructive action of the roots. Moreover, the most destructive plants are those with vegetative reproduction, since stolons and rhizomes cause the infesting plants to increase in size and propagate over large areas [13]. Table 3 summarizes some alterations produced by lichens, mosses, and higher plants [20,40].

During maintenance operations on historic buildings and archaeological sites, cleaning operations must be combined to remove colonizing vegetation, because their frequency or absence influences the type and degree of colonization by the plants. Indeed, in a study carried out by Motti and Bonanomi [35] on four castles in Southern Italy, three situated in the Naples metropolitan area (Maschio Angioino, Castel dell’Ovo, and Castel Sant’Elmo) and one in the Phlegraean Fields in the Municipality of Baia (Aragonese Castle of Baia), differences were observed in the analyzed plants due strictly to cleaning. For instance, Castel dell’Ovo had the lowest HI with a significant cover of *Helichrysum litoreum*, *Lactuca saligna,* and *Lobularia maritima*, delineating a different flora compared to Maschio Angioino and Castel Sant’Elmo. This floristic pattern could be related to the frequent cleaning of the wall surfaces combined with the spatial proximity to the sea. On the other hand, Maschio Angioino, although subject to periodical cleaning, is the only castle with very dangerous species such as *Ailanthus altissima*, *Ficus carica*, *Dittrichia viscosa,* and *Capparis spinosa.* This condition may be explained by the difficulty of reaching some areas of the castle during ordinary cleaning practices. Moreover, according to Dabghi et al. [62], the archaeological site of Volubilis in Morocco presents an undeniable biodeteriogenic action for careless cleaning. Beyond the mechanical and chemical breakdown of Volubilis material due to woody plants (*Ceratonia siliqua*, *Ficus carica*, *Olea europea*, etc.), therophytes such as *Diplotaxis catholica*, *Papaver rhoeas*, *Fumaria capreolata,* and *Avena sterilis* are not the least harmful plants because of their abundance at the site. The high abundance of these species could be explained by operations being carried out only occasionally at the site.

There are many control methods to reduce or avoid the risk of colonization, which depends on the type of plant, the structure of the building, its state of conservation, and its location. For herbaceous plants, the surest method of control with long-term advantages is the total removal of the plants, including their roots, and thereafter filling the cracks so that other seeds do not germinate in them. However, this method has limitations for ligneous plants due to the difficulty of removing the roots, and when the walls are impervious or very high. In this way, more complex methods must be used, including killing the plant with chemicals, or, if the position of the plant in the building permits, removing stones, extirpating the roots, and rebuilding. Chemical herbicides are more efficient than manual weeding, but their application depends on environmental (climate, chemical, and physical properties of materials, etc.) and toxicological aspects (toxicity, volatility, and biodegradability). Obviously, chemicals should not be used indiscriminately in towns as problems of elimination could arise with already polluted air [13].

## 5. Materials and Methods

For this review, a total of 62 articles were collected, focusing on the biodeterioration and colonization of stone monuments. We used the main international databases, such as Elsevier Journal Finder (https://elsevier.com, accessed on 10 November 2022), Scopus (https://scopus.com, accessed on 10 November 2022), and Google Scholar (https://scholar.google.com, accessed on 7 November 2022), with various combinations of keywords, e.g., “biodeterioration”, “biodeteriogenic plants”, “monument conservation” and “higher plants deterioration”. Further articles and books were identified from the references of these papers. Table 4 provides a summary of the articles in which case studies refer to higher plants, mosses, and lichens.

## 6. Conclusions

Stone cultural heritage sites and relics are commonly subjected to biochemical weathering [31]. Characterization, interpretation, rating, and prediction of weathering damage on stone monuments through precise diagnosis is necessary to preserve cultural heritage. In agreement with Fitzner and Heinrichs [45], a precise scientific procedure for the study and evaluation of damage may be monument mapping, which provides a detailed classification scheme of weathering forms. This evaluation, describing damage categories and indices, gives important information on: biodeterioration’s dependence on lithotypes and environmental influences, the factors and processes of stone weathering, and stone durability. The results obtained from monument mapping represent an important execution of efficient and economic monument preservation measures.

As previously discussed, a way to avoid or limit biodeterioration is to adopt preventive strategies before any damage becomes irreparable. It is often the case that the walls of historic buildings are colonized by plants already present in the surrounding area. For this reason, it is advisable to choose species for planting that conform to certain criteria, e.g., plants that should not invade the ruins, plants native to the surroundings, and plants that do not cause allergies. Simultaneously, as animals also contribute to seed dispersal, zoologists participate in maintenance measures mainly to limit bird fauna from roosting on monuments. The bird excrement causes corrosion, aesthetic damage, dispersal of seeds from wall vegetation, and it contributes to the formation of substrates for plant growth [13].

Based on studies of different archaeological sites and historical buildings, it was found that the invasion of non-native harmful species has been constantly progressing in recent decades. So, the spread of invasive trees represents a highly complex problem, particularly in urban systems, which requires new finding for the conservation of certain areas. However, obstacles of various aspects often appear during the operation and maintenance of sites. Since heritage sites are also a place of refuge for many plant species, there is a need to choose between the protection of monuments and the conservation of biodiversity, also considering the bioprotective action exerted by certain species acting as a barrier against weathering and thermal stress. Another issue depends on the cultural connections that people have with plants, particularly in cities. Indeed, local inhabitants often raise concerns when trees are to be removed or even appreciate the iconic value of the plants covering the monuments [7].

Further research and solutions need to be implemented to prevent and eliminate signs of biodeterioration. To address all of the issues and necessities related to the proper management of cultural heritage sites, new strategies must integrate multidisciplinary approaches considering the wide range of interests involved in heritage sites, including first of all the conservation of the monuments themselves [7]. Moreover, to reveal the effective importance of the ecology of plants colonizing walls considering all of the environmental and climatic factors, further studies are needed to better understand the relationship between plants and substrates and to characterize the above—as well as below—ground microclimate experienced by plants.

## Figures and Tables

**Table 1:** Comparison of the engineering properties of examined stones ([21], modified).

Properties	Stones			
	Ignimbrite	Tuff	Travertine	Andesite
	Min-Max	Min-Max	Min-Max	Mean
Water absorption	20.45–24.11	11.04–18.37	1.54–2.56	4.89
w_a_ (%)				
Effective porosity	29.91–33.18	18.67–27.10	3.88–6.10	10.79
n_e_ (%)				
Uniaxial comp. Strength	5.74–19.65	12.76–20.43	39.20–53.00	47.75
σ_c_ (MPa)				
Bohme abrasion value	37.89–56.28	21.20–36.68	14.55–30.13	14.77
ΔV (cm³/50 cm²)				

**Table 2:** Surface roughness values of examined stones. The roughness parameters Ra and Rq express the average and root mean square roughness, respectively ([21], modified).

Surface Roughness	Stones			
	Ignimbrite	Tuff	Travertine	Andesite
Ra (μm)	290.50 ± 8.12	95.80 ± 4.45	1109.98 ± 11.10	85.20 ± 3.70
Rq (μm)	372.98 ± 7.26	136.90 ± 4.69	1426.23 ± 12.05	120.69 ± 4.12

**Table 3:** Phenomenology of biological alterations in stone monuments ([20,40], modified).

Organism	Alteration
*Lichens*	Crusts, patches, pitting
*Mosses and liverworts*	Discoloration, green-gray patches
*Higher plants*	Cracks, collapse, detachment of material

**Table 4:** List of studies concerning plant identification and the assessment of hazard index ratings to describe biodeterioration processes and the influencing factors. The corresponding site, organism, method, and reference are reported for each study.

Site	Organism	Method	Reference
Forum of Baelo Claudia (Cadiz, Spain)	*Lichens*	Identification of lichens on sandstones and study of biodeterioration vs. bioprotection mechanisms.	Ortega-Calvo et al., 1995
Ancient monuments in Rome	Trees and shrubs: *Acer negundo*, *Ailanthus altissima*, *Ligustrum lucidum*, *Platanus hispanica*, *Robinia pseudoacacia*, *Capparis spinosa*	Patterns of distribution and spread of invasive plants by analyzing plant form, plant height, and hazard index.	Grapow and Ricotta, 2021
Archaeological site of Volubilis (Morocco)	Entire vegetation: *Foeniculum vulgare*, *Scorpiurus muricatus*, *Pallenis spinosa*, *Anchusa italica*, *Diplotaxis tenuifolia*, *Ficus carica*, etc.	Floristic diversity and analysis of its biodeteriogenic effect by identification of species.	Dabghi et al., 2021
Fortress of Mazagan (Morocco)	All the plants at the base of walls: *Chenopodium murale*, *Senecio vulgaris*, *Euphorbia terracina*, *Olea europaea*, *Nicotiana glauca*, etc.	Identification of plant species and analysis of its potential effects on the substrate and monuments.	Dahmani et al., 2020
Pasargadae World Heritage Site (Iran)	Entire vegetation: *Medicago sativa*, *Lactuca serriola*, *Lolium perenne*, *Poa bulbosa*, *Fumaria parviflora*, *Galium setaceum*, etc.	Floristic identification, general abundance of each species, and risk evaluation.	Hosseini and Caneva, 2021
Walls on Hong Kong Island	Tree species: *Ficus microcarpa*, *Celtis sinensis*, *Broussonetia papyrifera*, *Morus alba*, *Ficus elastica*, *Bombax ceiba*, etc.	Multiple regression analysis to examine the relationship between habitat factors and vegetation occurrence.	Jim and Chen, 2010
Embankments along the Tiber River, the Lungotevere (Rome)	Entire vegetation: *Parietaria judaica*, *Sonchus tenerrimus*, *Geranium rotundifolium*, *Vicia sativa*, *Dittrichia viscosa*, *Ailanthus altissima,* etc.	Species identification and biodeterioration assessment by hazard index rating.	Kumbaric et al., 2012
Three Historical Calabrian (Southern Italy) Churches	Entire vegetation: *Ailanthus altissima*, *Anthemis arvensis*, *Ficus carica*, *Sambucus nigra*, *Trifolium pratense*, *Veronica polita*, etc.	Species identification and biodeterioration assessment by hazard index rating.	Mascaro et al., 2021
Four Castles in Campania (Southern Italy)	Entire vegetation: *Ailanthus altissima*, *Capparis spinosa*, *Daucus carota*, *Lobularia maritima*, *Reichardia picroides*, *Sonchus oleraceus,* etc.	Species identification and biodeterioration assessment by hazard index rating. Floristic similarities by a multivariate cluster analysis.	Motti and Bonanomi, 2018
Royal Palace of Portici (Southern Italy)	Entire vegetation: *Acer negundo*, *Artemisia annua*, *Celtis australis*, *Dittrichia viscosa*, *Quercus ilex*, *Verbena officinalis*, etc.	Species identification and biodeterioration assessment by hazard index rating.	Motti and Stinca, 2011
Phlegraean Fields Archaeological Park (Southern Italy)	Entire vegetation: *Erigeron sumatrensis*, *Sonchus tenerrimus*, *Parietaria judaica*, *Capparis spinosa*, *Pistacia lentiscus*, *Ficus carica*, etc.	Species identification and biodeterioration assessment by hazard index rating. Comparison of plant communities by cluster analysis based on Bray–Curtis similarity distance	Motti et al., 2020
Villa Rufolo (Southern Italy)	Entire vegetation: *Ficus carica*, *Hedera helix*, *Capparis orientalis*, *Parthenocissus tricuspidata*, *Centranthus ruber*, *Parietaria judaica*, etc.	Species identification and biodeterioration assessment by hazard index rating.	Motti et al., 2021
Bridges over Zayandeh-Rood River (Iran)	Mosses, algae, and higher plants: *Trichostomum* sp., *Gymnostomum* sp., *Fissidens bryoides*, *Cinclidotus* sp., *Ficus carica*, *Salix* sp., etc.	Taxa identification and chemical analysis of moss samples.	Shirzadian and Uniyal, 2008

## Data Availability

Data are available upon request.
